# Trends in tuberculosis notification and mortality and factors associated with treatment outcomes in Serbia, 2005 to 2015

**DOI:** 10.2807/1560-7917.ES.2020.25.1.1900322

**Published:** 2020-01-09

**Authors:** Maja Stosic, Sandra Sipetic Grujicic, Anita Grgurevic, Vesna Kuruc, Lidija Ristic, Gordana Antonijevic, Miroslav Jevtic, Dragana Plavsa, Tatjana Adzic Vukicevic

**Affiliations:** 1 Public Health Institute of Serbia “Dr Milan Jovanovic Batut”, Belgrade, Serbia; 2 Institute of Epidemiology, Faculty of Medicine, University of Belgrade, Belgrade, Serbia; 3 Institute for Pulmonary Diseases of Vojvodina, Sremska Kamenica, Serbia; 4 Clinic for Pulmonary Diseases, Clinical Center Nis, Nis, Serbia; 5 Special Hospital for Pulmonary Diseases Ozren-Sokobanja, Sokobanja, Serbia; 6 University Clinic for Pulmonology, Clinical Center of Serbia, Belgrade, Serbia

**Keywords:** Tuberculosis, Serbia, notification, mortality, factors, treatment outcomes

## Abstract

**Background:**

Previously a country with medium tuberculosis (TB) burden, Serbia almost reached a low TB burden during the period 2005 to 2015.

**Aim:**

The aim of this study was to analyse the trends in notification rates and treatment success rates as well as to identify predictors of treatment outcomes.

**Methods:**

We performed a trend analysis and logistic regression analysis of 17,441 TB cases registered from 2005 to 2015 in all health facilities in Serbia, to identify predictors of treatment success, loss to follow-up and mortality.

**Results:**

From 2005 to 2015, TB notification rate and mortality in Serbia decreased but treatment success remained below the global target. Loss to follow-up was associated with retreatment (odds ratio (OR) = 2.38; 95% confidence interval (CI): 2.08–2.77), male sex (OR = 1.57; 95% CI: 1.39–1.79), age younger than 65 years (OR = 1.37; 95% CI: 1.20–1.51), lower education level (OR = 2.57; 95% CI: 1.74–3.80) and pulmonary TB (OR = 1.28; 95% CI: 1.06–1.56). Deaths were more frequent in retreatment cases (OR = 1.39; 95% CI: 1.12–1.61), male patients (OR = 1.34; 95% CI: 1.19–1.52), those 65 years and older (OR = 4.34; 95% CI: 4.00–5.00), those with lower education level (OR = 1.63; 95% CI: 1.14–2.33) and pulmonary TB (OR = 2.24; 95% CI: 1.78–2.83).

**Conclusions:**

Special interventions should be implemented to address groups at risk of poor treatment outcome.

## Introduction

Tuberculosis (TB) is still one of the major public health problems affecting more than 10 million people and causing 1.6 million deaths worldwide [1]. Since 1994, milestones of TB prevention and control have been the three World Health Organization (WHO) strategies Directly observed treatment short course (DOTS), Stop TB and End TB [2,3]. Implementing the WHO strategies, many countries established between 2000 and 2014 the basic requirements for providing high-quality TB diagnosis and treatment [3]. Those strategies focused on case notification and monitoring of treatment outcome as the essential measures to evaluate the effectiveness of interventions and identify potential gaps in TB control [4]. The global target is to reach a treatment success rate (TSR) of at least 85% [2]. However, the WHO's post-2015 End TB Strategy aims for at least 90% [3]. TB disproportionately affects different segments of the population. Higher male-to-female ratio was reported in some studies and surveillance data as well as higher TB notification rate and lower mortality rate among males [1,5,6]. Changes in the occurrence of the disease by age, sex, education, anatomical site of the disease, history of previous treatment, HIV status and drug resistance are also important indicators of TB control programmes [7-11].

In the period 2005–15, Serbia almost became a country with a low TB burden, reducing TB notification rate from 32 per 100,000 in 2005 to 13 per 100,000 in 2015 by implementing the WHO DOTS and Stop TB strategies in the National Tuberculosis Programme with financial support from the Global Fund to Fight AIDS, Tuberculosis and Malaria (GFATM). An analysis of trends and differences in TB notification and treatment outcomes by sex, age, education, occupation, TB localisation and history of previous treatment has not yet been performed in Serbia. It may help improve our understanding of the performance of the TB control programme. Previous studies in Serbia have focused on trends in TB incidence and mortality and characteristics of TB among the elderly population [12-14]. However, these studies did not analyse factors associated with successful and unsuccessful treatment outcome, which could provide useful evidence for targeted evidence-based interventions. Therefore, the objective of this study was to assess the trends in TB notification, TB mortality rate and treatment success and to identify factors associated with treatment success, loss to follow-up and mortality among TB patients notified in Serbia in a period of 11 years.

## Methods

We performed retrospective trend analysis and analysis of treatment outcomes of all (n = 17,441) TB cases notified in Serbia in the period 2005–15 from all health facilities, based on the electronic data collection of the Ministry of Health and Institute of Public Health of Serbia. The electronic case-based data collection system in Serbia was introduced in 2005 with the support of the GFATM in line with the WHO TB notification form. Standard indicators from the WHO checklist S*tandards and benchmarks for TB surveillance and vital registration systems* were used to assess internal consistency of the data: changes over time in TB notification rate, the ratio between the number of notified pulmonary and extra-pulmonary TB cases, and the male-to-female ratio of TB cases [15]. 

To describe TB epidemiology in the country, we analysed notified TB cases by age, sex, education, occupation, anatomical site of the disease, history of previous treatment, HIV status (recorded since 2010) and multidrug resistance. Treatment results included: cured, treatment completed, defaulted (lost to follow-up), died, failed and not evaluated [16,17]. National TB control guidelines for Serbia, in line with WHO guidelines, were used for TB diagnosis and case definition [16].

### Tuberculosis diagnosis

TB diagnosis was based on clinical and radiological findings and was confirmed bacteriologically and/or histologically. Laboratory confirmation was performed by detection of *Mycobacterium tuberculosis* complex from a clinical specimen, either by culture or by a newer molecular technique [18].

### Case definition and classification

*Pulmonary tuberculosis* (PTB) refers to any bacteriologically confirmed or clinically diagnosed case of TB involving the lung parenchyma or the tracheobronchial tree. PTB also includes cases that affect the lung with additional extra-pulmonary manifestations.

*Extra-pulmonary tuberculosis* (EPTB) refers to any bacteriologically confirmed or clinically diagnosed case of TB involving organs other than the lungs, e.g. pleura, lymph nodes, abdomen, genitourinary tract, skin, joints and bones or meninges.

*New patients* are defined as those who have never been treated for TB or have taken anti-TB drugs for less than 1 month.

*Previously treated* patients are defined as those who have received 1 month or more of anti-TB drugs in the past.

*Multidrug resistance* refers to resistance to at least isoniazid and rifampicin together.

Treatment outcomes were categorised according to the latest WHO definitions and reporting framework for tuberculosis [11].

### Statistical analysis

Descriptive statistics of trends of TB notification, TB mortality rates and treatment outcomes during the 11-year study period were performed for all PTB and EPTB cases. Population data for each year were obtained from the National Statistical Office [18]. We analysed trends of case notification by age, sex, education, occupation, anatomical site of the disease, history of previous treatment, HIV status and first-line drug susceptibility.

A logistic regression analysis was used to identify factors associated with treatment success, loss to follow-up and mortality. We used three separate models. The variables age, sex, education, occupation, anatomical site of the disease, history of previous treatment, HIV status and first-line drug susceptibility test (DST) were entered in each univariate logistic regression analysis (ULRA) model. For the first model, the outcome was dichotomised as treatment success (cured or completed) vs unsuccessful treatment (failed, died, lost to follow-up or not evaluated). For the second model, the outcome was dichotomised as lost to follow-up vs follow-up (treatment success, failed, died, or not evaluated). For the third model, the outcome was dichotomised as died vs not died (treatment success, failed, lost to follow-up or not evaluated).

The variables that showed statistically significant association at p value ≤ 0.05 in ULRA were entered in multivariable logistic regression analysis (MLRA) models. Data were analysed using the Statistical Package for Social Sciences (IBM SPSS) version 24 and p values ≤ 0.05 were considered statistically significant.

### Ethical statement

This study was approved by the Ethics Committee of the Institute of Public Health of Serbia (No 24/2016). Personal identifiers of TB cases were coded to maintain the confidentiality of the patient information before analysis, and patient records or information were anonymised and de-identified before analysis.

## Results

### Internal consistency indicators

The mean percentage of annual changes of TB notification rate from 2005 to 2015 was 8.25%. During the same period, the ratio of nationally notified PTB vs EPTB cases ranged from 6.04 to 8.09, with a slight decrease in PTB cases from 2013 to 2015. The male-to-female ratio of notified TB cases was generally consistent (range: 1.40–1.62), but there was a notable increase in the proportion of male cases over time.

### Trends analysis of tuberculosis notifications

**Figure 1:**
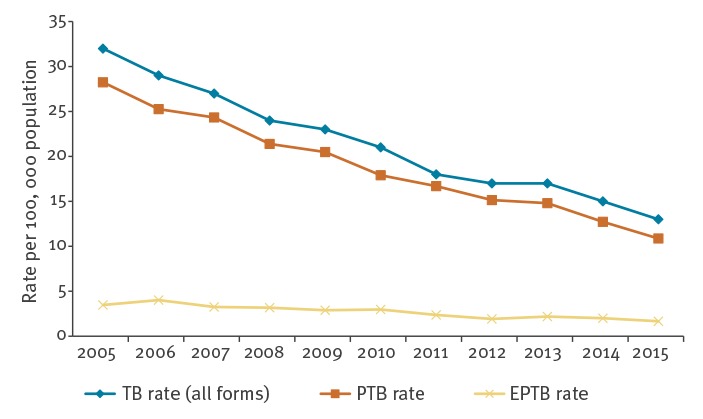
Trends in tuberculosis notification rates per 100,000 population by year and by anatomical site of the disease, Serbia, 2005–2015 (n = 17,441)

EPTB: Extra-pulmonary tuberculosis; PTB: pulmonary tuberculosis; TB: tuberculosis.

The ТB notification rate for all forms of TB decreased significantly between 2005 and 2015, from 32 per 100,000 population in 2005 to 13 per 100,000 in 2015 (Table 1, Figure 1) (p value for trend < 0.001). Likewise, we observed a significant decrease in the PTB notification rate, from 28 per 100,000 population in 2005 to 11 per 100,000 in 2015 (p value for trend < 0.001), while there were no great variations in the EPTB notification rate (p value for trend > 0.05). Over the observed period, male-to-female ratio and notification rates per age category remained unchanged (p value for trend > 0.05). 

**Table 1:** Characteristics of notified tuberculosis cases, Serbia, 2005–2015 (n = 17,441)

Characteristics	2005		2006		2007		2008		2009		2010		2011		2012		2013		2014		2015		Total
	n	%	n	%	n	%	n	%	n	%	n	%	n	%	n	%	n	%	n	%	n	%	
All cases	2,378	14	2,170	12	2,063	12	1,831	10	1,712	10	1,523	9	1,379	8	1,228	7	1,217	7	1,051	6	889	5	17,441
Sex																							
Male	1,444	61	1,328	61	1,256	61	1,110	61	1,045	61	915	60	834	60	758	62	753	62	607	58	541	61	10,591
Female	934	39	842	39	807	39	721	39	667	39	608	40	545	40	470	38	464	38	444	42	348	39	6,850
Age category (years)																							
0–14	20	1	24	1	29	1	22	1	21	1	17	1	13	1	18	1	14	1	21	2	8	1	207
15–24	180	8	169	8	143	7	148	8	135	8	138	9	96	7	101	8	93	8	77	7	57	6	1,337
25–34	258	11	234	11	206	10	183	10	162	9	146	10	151	11	143	12	136	11	101	10	113	13	1,833
35–44	340	14	283	13	294	14	244	13	238	14	226	15	167	12	170	14	172	14	173	16	126	14	2,433
45–54	465	20	441	20	432	21	371	20	299	17	260	17	272	20	220	18	201	17	159	15	169	19	3,289
55–64	338	14	289	13	308	15	306	17	300	18	244	16	258	19	223	18	219	18	192	18	156	18	2,832
≥ 65	777	33	730	34	651	32	557	30	557	33	492	32	422	31	353	29	382	31	328	31	260	29	5,509
Education																							
High school or lower	2,291	96	2,057	95	1,944	94	1,732	95	1,622	95	1,442	95	1,294	94	1,164	95	1,156	95	975	93	812	91	16,489
University or higher	87	4	113	5	119	6	99	5	90	5	81	5	85	6	64	5	61	5	76	7	77	9	952
Occupation																							
Unemployed	220	9	262	12	304	15	292	16	311	18	316	21	285	21	304	25	303	25	200	19	161	18	2,958
Employed	2,158	91	1,908	88	1,759	85	1,539	84	1,401	82	1,207	79	1,094	79	924	75	914	75	851	81	728	82	14,483
Anatomical site of the disease																							
PTB	2,118	89	1,872	86	1,820	88	1,594	87	1,500	88	1,306	86	1,208	88	1,090	89	1,061	87	908	86	771	87	15,248
EPTB	260	11	298	14	243	12	237	13	212	12	217	14	171	12	138	11	156	13	143	14	118	13	2,193
History of previous treatment																							
New patient	2,118	89	1,884	87	1,772	86	1,567	86	1,502	88	1,343	88	1,240	90	1,090	89	1,093	90	952	91	800	90	15,361
Previously treated	260	11	286	13	291	14	264	14	210	12	180	12	139	10	138	11	124	10	99	9	89	10	2,080
HIV status^a^																							
Known	NA		NA		NA		NA		NA		4	0	67	5	95	8	118	10	124	12	90	10	498
Positive	NA		NA		NA		NA		NA		4	0	6	0	4	0	17	1	8	1	4	0	43
Unknown	NA		NA		NA		NA		NA		1,515	99	1,306	95	1,129	92	1,082	89	919	87	795	89	6,746
Multidrug resistance																							
No	181	8	592	27	865	42	973	53	1,074	63	1,092	72	954	69	863	70	811	67	674	64	508	57	8,587
Yes	15	1	15	1	33	2	15	1	11	1	13	1	7	1	7	1	8	1	11	1	5	1	140
Unknown	2,182	92	1,563	72	1,165	56	843	46	627	37	418	27	418	30	358	29	398	33	366	35	376	42	8,714

EPTB: Extra-pulmonary tuberculosis; NA: not available; PTB: pulmonary tuberculosis.

^a^ Data on HIV status are available in the TB register from 2010 [19].

The proportions of multidrug-resistant TB cases remained stable at 1–2% over time (p value for trend > 0.05). The proportions of negative DST results increased significantly from 7.6% in 2005 to 57.1% in 2015. There was also a significant decrease in unknown DST results from 91.7% in 2005 to 42.3% in 2015 (p value for trend < 0.05).

The proportions of HIV-positive TB cases remained stable in the period from 2010 to 2015 (p value for trend > 0.05), while the proportions of HIV-negative results increased significantly (p value for trend < 0.05), from 0.3% in 2010 to 10.1% in 2015.

### Trends in treatment outcomes

Among the 17,441 notified cases, treatment outcome was available for 17,388. Over the 11-year period, there were no significant changes in the treatment outcomes of drug-susceptible TB (Table 2). Treatment success slightly increased among all new TB cases (p value for trend > 0.05), from 83% in 2005 to 84% in 2015, while it slightly decreased among retreatment cases (p value for trend > 0.05), from 70% in 2005 to 67% in 2015. Among new cases, the increase in treatment success was slightly higher among EPTB cases (p value for trend > 0.05), from 85% in 2005 to 90% in 2015. The proportion of patients lost to follow-up decreased slightly, from 6% in 2005 to 5% in 2015 among all new cases (p value for trend > 0.05) and from 14% in 2005 to 13% in 2015 among retreatment cases (p value for trend > 0.05).

**Table 2:** Treatment outcomes among tuberculosis cases, Serbia, 2005–2015 (n = 17,388)

Treatment outcome	2005		2006		2007		2008		2009		2010		2011		2012		2013		2014		2015		Total
	n	%	n	%	n	%	n	%	n	%	n	%	n	%	n	%	n	%	n	%	n	%	
New pulmonary TB																							
Cured + completed	1,541	82	1,330	83	1,305	85	1,164	85	1,122	86	981	86	912	84	784	82	751	79	659	80	574	83	11,123
Lost to follow-up	118	6	95	6	71	5	79	6	62	5	54	5	50	5	52	5	56	6	34	4	37	5	708
Died	105	6	115	7	106	7	93	7	82	6	77	7	96	9	90	9	87	9	90	11	56	8	997
Failed	15	1	21	1	19	1	27	2	15	1	10	1	5	0	7	1	10	1	7	1	9	1	145
Not evaluated	94	5	47	3	41	3	12	1	24	2	17	1	17	2	24	3	43	5	37	4	16	2	372
Total	1,873		1,608		1,542		1,375		1,305		1,139		1,080		957		947		827		692		13,345
New extra-pulmonary TB																							
Cured + completed	216	88	244	88	211	92	199	94	177	79	184	90	140	88	109	82	119	82	118	84	106	93	1,823
Lost to follow-up	12	5	15	5	10	4	8	4	8	4	8	4	5	3	16	12	9	6	6	4	6	5	103
Died	10	4	9	3	8	3	3	1	3	1	6	3	12	8	5	4	8	5	9	6	1	1	74
Failed	0	0	0	0	0	0	0	1	1	0	2	1	0	0	0	0	1	1	0	0	0	0	4
Not evaluated	7	3	8	3	1	0	1	0	35	16	4	2	3	2	3	2	9	6	7	5	1	1	79
Total	245		276		230		211		224		204		160		133		146		140		114		2,084
All new TB cases																							
Cured + completed	1,757	83	1,574	84	1,516	86	1,363	86	1,299	85	1,165	87	1,052	85	893	82	870	80	777	80	680	84	12,946
Lost to follow-up	130	6	110	6	81	5	87	5	70	5	62	5	55	4	68	6	65	6	40	4	43	5	811
Died	115	5	124	7	114	6	96	6	85	6	83	6	108	9	95	9	95	9	99	10	57	7	1071
Failed	15	1	21	1	19	1	28	2	16	1	12	1	5	0	7	1	11	1	7	1	9	1	150
Not evaluated	101	5	55	3	42	2	13	1	59	4	21	2	20	2	27	2	52	5	44	5	17	2	451
Total	2,118		1,884		1,772		1,587		1,529		1,343		1,240		1,090		1,093		967		806		15,429
Previously treated cases																							
Cured + completed	174	70	187	76	202	72	174	73	154	75	125	77	106	79	95	70	87	71	65	64	58	67	1,427
Lost to follow-up	34	14	25	10	46	16	20	8	26	13	17	10	12	9	22	16	13	11	13	13	11	13	239
Died	26	10	24	10	23	8	29	12	19	9	16	10	9	7	13	10	8	7	11	11	10	12	188
Failed	6	2	1	0	7	2	1	0	0	0	2	1	3	2	2	1	4	3	0	0	3	3	29
Not evaluated	8	3	8	3	4	1	13	5	6	3	2	1	5	4	3	2	11	9	12	12	4	5	76
Total	248		245		282		237		205		162		135		135		123		101		86		1,959
Multidrug-resistant TB cases^a^																							
Cured + completed	NA		NA		NA		NA		11	55	13	76	12	71	4	57	9	75	8	89	4	80	61
Lost to follow-up	NA		NA		NA		NA		1	5	1	6	2	12	0	0	1	8	0	0	1	20	6
Died	NA		NA		NA		NA		6	30	2	12	2	12	2	29	2	17	1	11	0	0	15
Failed	NA		NA		NA		NA		2	10	0	0	1	6	1	14	0	0	0	0	0	0	4
Not evaluated	NA		NA		NA		NA		0	0	1	6	0	0	0	0	0	0	0	0	0	0	1
Total^b^	NA		NA		NA		NA		20		17		17		7		12		9		5		87

MDR: multidrug-resistant; NA: not available; TB: tuberculosis.

^a^ Treatment outcome of MDR TB cases is available in the TB register from 2009, the year when organised MDR TB treatment started in Serbia.

^b^ The number of laboratory-confirmed MDR TB cases enrolled in second-line TB treatment. The difference between registered patients and patients enrolled in treatment is due to backlog of patients from previous years.

**Figure 2:**
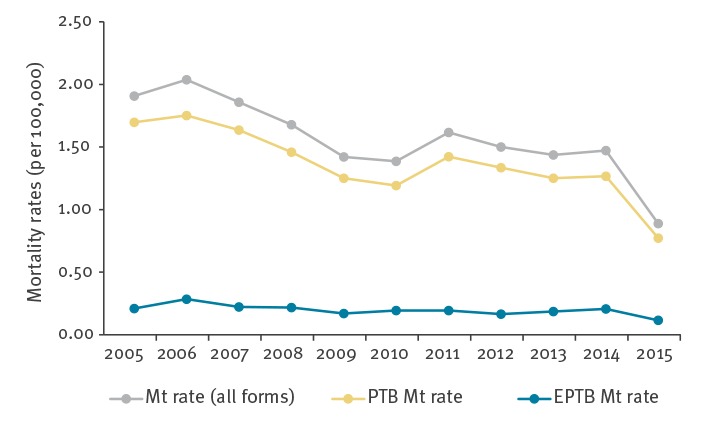
Trends of tuberculosis mortality rates per 100,000 population by year and by anatomical site of disease, Serbia, 2005–2015 (n =1,259)

EPTB: Extra-pulmonary tuberculosis; Mt: mortality; PTB: pulmonary tuberculosis.

The ТB mortality rate for all forms of TB was twice reduced between 2005 and 2015, from 1.91 per 100,000 population in 2005 to 0.89 per 100,000 in 2015 (Figure 2) (p value for trend < 0.05). This was due to a significant decrease in PTB mortality rate, from 1.70 per 100,000 population in 2005 to 0.77 per 100,000 in 2015 (p value for trend < 0.05), while there was no great variation in EPTB mortality rate (p value for trend > 0.05).

Treatment success rate of multidrug-resistant (MDR) TB cases increased significantly from 55% in 2009 to 80% in 2015 (p value for trend < 0.05). These data correspond to the period 2009–15, because organised treatment of MDR TB in Serbia started in 2009 (Table 2).

### Characteristics of the cases

Of the 17,441 cases, 6,850 (39.3%) were women and 10,591 (60.7%) were men, yielding a male-to-female ratio of 1.5∶1. The mean age for all cases was 46 years (standard deviation (SD): 17), 44 years for men (SD: 16) and 49 years for women (SD: 18) years. Most of the cases, 15,248 (87.4%), had PTB while 2,193 (12.6%) had EPTB. A total of 16,489 (94.5%) cases had finished high school or a lower level of education, whereas 952 (5.5%) had completed university level or higher. Most of the cases were employed (n = 14,483; 83.0%). Most were new cases (n = 15,361; 88.0%), while 2,080 (12.0%) were previously treated (Table 1). There were 140 (1.7%) notified and recorded MDR cases among 8,437 cases (48.4%) covered by DST, while for 9,004 cases (51.6%), DST results were unknown (Table 1).

Data on HIV status have been recorded in the TB register since 2010 and 7,287 TB cases were notified in the period from 2010 to 2015. There were 43 (7.9%) notified HIV-positive cases among 541 tested and recorded, while for 6,746, the HIV status was unknown. 

MDR TB cases were included in each of the three univariate models. The variables that showed a statistically significant association at p value ≤ 0.05 in ULRA were entered in multivariable models. MDR TB was not statistically significantly associated with treatment success (model 1), loss to follow-up (model 2) or mortality (model 3) and was therefore not included in the models of MLRA.

### Factors associated with treatment outcomes

In the multivariable logistic regression analysis (Table 3), factors associated with treatment success were: new TB (OR = 2.05; 95% CI: 1.86–2.27), female sex (OR = 1.41; 95% CI: 1.30–1.51), age below 65 years (OR = 1.61; 95% CI: 1.49–1.74), university or higher level of education (OR = 1.62; 95% CI: 1.23–1.88) and EPTB (OR = 1.53; 95% CI: 1.36–1.74). A higher risk of being lost to follow-up was associated with previous TB treatment (OR = 2.38; 95% CI: 2.08–2.77), male sex (OR = 1.57; 95% CI: 1.39–1.79), age below 65 years (OR = 1.37; 95% CI: 1.20–1.51), high school or lower level of education (OR = 2.57; 95% CI: 1.74–3.80) and PTB (OR = 1.28; 95% CI: 1.06–1.56). In addition, a higher risk of TB mortality was related to previous TB treatment (OR = 1.39; 95% CI: 1.12–1.61), male sex (OR = 1.34; 95% CI: 1.19–1.52), age 65 years and above (OR = 4.34; 95% CI: 4.00–5.00), high school or lower level of education (OR = 1.63; 95% CI: 1.14–2.33) and PTB (OR = 2.24; 95% CI: 1.78–2.83).

**Table 3:** Factors associated with treatment success, loss to follow-up and mortality among notified tuberculosis cases, multivariable logistic regression analysis, Serbia, 2005–2015 (n = 17,441)

Characteristics	Treatment success		Lost to follow-up		Mortality	
	OR (95% CI)	p value	OR (95% CI)	p value	OR (95% CI)	p value
Sex: male (Ref: female)	0.71 (0.66–0.77)	0.000	1.57 (1.39–1.79)	0.000	1.34 (1.19–1.52)	0.000
Age category: 0–64 years (Ref: ≥ 65 years)	1.61 (1.49–1.74)	0.000	1.37 (1.20–1.51)	0.000	0.23 (0.20–0.25)	0.000
Education: high school or lower (Ref: university or higher)	0.66 (0.53–0.81)	0.000	2.57 (1.74–3.80)	0.000	1.63 (1.14–2.33)	0.000
Occupation: Unemployed (Ref: employed)	0.91 (0.80–1.03)	0.141	1.10 (0.92–1.31)	0.279	1.08 (0.89–1.31)	0.403
Anatomical site of the disease: PTB (Ref: EPTB)	0.65 (0.57–0.73)	0.000	1.28 (1.06–1.56)	0.010	2.24 (1.78–2.83)	0.000
History of previous treatment: new patient (Ref: retreatment)	2.05 (1.86–2.27)	0.000	0.42 (0.36–0.48)	0.000	0.73 (0.62–0.86)	0.000

CI: confidence interval; EPTB: Extra-pulmonary tuberculosis; OR: odds ratio; PTB: pulmonary tuberculosis; Ref: reference value.

## Discussion

From 2005 to 2015 in Serbia, annual changes in the TB notification rate were less than 10%. The ratio of PTB to EPTB cases notified nationally ranged from 6.04 to 8.09, with a slight decrease in PTB cases from 2013 to 2015, suggesting that these data were internally consistent [16]. The male-to-female ratio increasing over time may suggest women were less likely in the later years than in the earlier years to seek healthcare and/or were underdiagnosed when they did seek care. In addition, it may indicate that epidemiologically, there was a real increase in men (or decrease in women) who developed TB [20].

Over the 11-year study period, we found a decrease in TB notification and mortality rates and a stable trend in treatment outcomes and notification rates per age category, education, occupation, anatomical site of the disease and history of previous treatment. The dominant proportion of male TB cases corresponds to the global surveillance data [1] and surveillance data for Europe [5], with the male-to-female ratio ranging from 1.5∶1 to 2∶1. 

We found that the proportion of PTB among TB patients in our study was 20% higher than the proportion of PTB among TB patients globally [1] and in the European Union/European Economic Area (EU/EEA) [5]. Solovic et al. reported that the percentage of EPTB cases in the EU/EEA) in 2011 ranged from 4% to 48% [21]. The differences may be explained by different risk factors for EPTB or by diagnostic challenges as EPTB can manifest with a variety of symptoms. Low levels of suspicion of clinicians as well as difficulties in obtaining adequate samples for confirmation are also reported as a challenge in EPTB diagnosis [21]. In addition, some of the reasons for higher proportion of PTB in Serbia compared with EU/EEA countries could be due to low HIV prevalence in Serbia [22] and to the dominant proportion of men among TB patients as there is evidence that the incidence of EPTB is likely to be higher among women than men [23]. 

We found that the proportion of previously treated cases was 20 percentage points lower in Serbia than globally [1]. This could indicate good implementation of the DOTS strategy, good case management, low burden of drug-resistant TB, good adherence to treatment or good access to and availability of TB services [2]. We assume that reliability of the data can be considered satisfactory because TB case definitions are clearly laid out in the guidelines for TB doctors as a part of routine TB training in the country and are consistent with the WHO guidelines. Firstly, TB cases are usually classified at the moment of filling out the TB notification form, by a physician who diagnosed TB based on medical investigations and medical records. Data from the TB notification form are further validated at the regional level by epidemiologists and pulmonologists before they are entered into a case-based electronic data collection system. A third round of data quality control is usually performed at the central (national) level in the form of automatic checks for duplicates, missing records and consistency during the process of data cleaning before data analysis; the central level receives copies of all case-based TB notification forms.

Educational status of TB patients in our study was lower than in the general population in Serbia [24]. There were no data on educational status of TB patients in Europe. Nevertheless, a large body of scientific evidence supports education as an important determinant of health and indicates that lower education is associated with poor health, stress and lower self-esteem [25]. The majority of patients in our study were employed. In most European countries, there is a greater share of unemployment among TB patients [26].

The proportion of HIV-positive TB cases was consistent with reports from the Balkan countries (excluding Romania with significantly higher TB/HIV prevalence) [1,27] and lower than in other EU/EEA countries [5]. Studies conducted in Africa and Asia found a much higher prevalence of HIV infection among TB patients (30–38%) [28,29]. The slight increase in the proportion of HIV-positive TB cases observed in Serbia was due to implementation of the WHO policy on collaborative TB/HIV activities and increased HIV testing of TB patients. However, among drug users, homeless people, prisoners and alcoholics, the proportion of TB cases with known HIV status is still very low in Serbia compared with EU/EEA countries (68%) because HIV testing is not routinely offered to all TB patients. There is a need for continuous training of all medical and non-medical workers who deal with TB and HIV infection and for a joint TB/HIV communication and social mobilisation strategy [5,30]. Implementation of the WHO Stop TB strategy resulted in an increased DST coverage, in line with the EU countries [5]. Given that the latest WHO End TB strategy target is 100% DST coverage, this is a programmatic challenge to be addressed in the coming years. We identified very small percentages of notified TB cases among children (0.8–2% of notified cases per year were children aged 0–14 years) in relation to WHO standards and benchmarks for childhood TB (5–15%) for middle-income countries where Serbia belongs [20]. This result pointed to under-reporting because of limited sensitivity of surveillance system as well as limited capacities of the TB programme to diagnose childhood TB.

The treatment success rate in Serbia was higher than the EU treatment success rate [5] but still below the global target of 90% [3]. The minor improvements during the study period could be attributed to better overall case management (early diagnosis and treatment, reduction in loss to follow-up and mortality). Other possible reasons could be community-based patient support interventions which improved access to and use of TB control services and decreased the proportion of non-evaluated cases compared with previous years [3].

We identified previous TB treatment, male sex, lower level of education and PTB as independent predictors of loss to follow-up and mortality, consistent with many other studies [29,31]. Some studies identified unemployment [32] and migration [33] as predictors of loss to follow-up caused by poor adherence to treatment. Although there were no foreign-born cases registered in our study, there is a possibility of under-reporting because of the lack of a sensitive surveillance system. Authors from the United Kingdom accentuated the public health implications of patients lost to follow-up, who may contribute to ongoing disease transmission and the risk of drug resistance [32]. Patients with severe forms of extra-pulmonary disease may be less likely to become lost, because they are likely to be in closer contact with the healthcare provider and may be hospitalised [34]. Weaknesses in the health system, such as lack of updated clinical guidelines, non-compliance of healthcare professionals with the guidelines, interruptions in drug supply or poor training, supervision and organisation of TB programmes, have also been identified as factors contributing to the poor management of patients, resulting in treatment interruptions and loss to follow-up [2,23].

We found age 65 years and above to be an independent predictor of TB mortality. Many studies reported higher mortality rates among elderly people [4,12]. Co-morbidities among elderly people influence the reactivation of previously acquired TB disease by reducing the immunity. Moreover, immunocompromised patients are more likely to develop severe disseminated forms of TB disease and adverse drug reactions [12,34]. Delay in diagnosis and treatment among older age groups was also identified, which could increase the risk of death. In addition, we identified age below 65 years as predictor of loss to follow-up. Patients younger than 65 years are more likely to be immunocompetent, with fewer co-morbidities. Loss to follow-up among this group of patients may be related to social barriers and perception of stigma. TB is known to be a highly stigmatised disease and many studies have identified stigma as an obstacle in cooperation and communication with the health service, resulting in delayed seeking of medical help, and an obstacle during treatment, leading to interruptions, failure, recurrence and drug resistance [34,35].

### Limitations

We could not distinguish between deaths that were due to TB or deaths that were due to other diseases during TB treatment because this information was not available. It was not possible to investigate other factors associated with TB treatment outcomes, such as socioeconomic factors, tobacco use, drug or alcohol abuse, other co-morbidities or homelessness. Data on DST results were missing for half of the cases, some of whom may have had MDR TB. Despite its limitations, our study provides evidence on the risk factors associated with TB treatment outcomes in Serbia. Further analyses are needed to explore further associations.

## Conclusions

In order to improve performance of the TB programme in Serbia, it is necessary to address groups at risk of poor treatment outcomes. To prevent loss to follow-up and mortality and to achieve better adherence to treatment, case management has to be improved towards providing holistic patient-centred inter-sectoral interventions and support during treatment. It is important to update the national TB prevention and control strategy and to revise existing guidelines in line with the available scientific evidence, so as to increase coverage of DST and HIV testing and improve early detection of HIV co-infection and drug resistance, the main obstacles in modern TB control.
